# The Cure of the Social Evil

**Published:** 1906-11

**Authors:** 


### EDITORIAL NOTES AND COMMENTS.

The Business office of THE JOURNAL-RECORD is 1007-1008 Century Building.

The Editorial office is 318-319-320 The Prudential.

Address all Business communications to Dr. M. B. Hutchins, Manager.

Make remittances payable to THE ATLANTA JOURNAL-RECORD OF MEDICINE.

On matters pertaining to the Editorial and Original Communications address Dr. Bernard Wolff, Atlanta.

Reprints of original articles will be furnished at cost price. Requests for the same should always be made *on the manuscript*.

We will present, post-paid, on request, to each contributor of an original article, twenty (20) marked copies of THE JOURNAL-RECORD containing such article.

### THE CURE OF THE SOCIAL EVIL.

THE *Journal of the American Medical Association*, in a late number, contains a group of papers upon the social evil and how best to suppress it. The chief point in all the papers appears to be an agreement that the proper methods to pursue to mitigate the evil lie in educating the public in the nature of venereal disease and the perils that surround unwary youth.

Information of this character should be given out as a phase of pedagogics and be included among the multifarious duties that doctors owe to the public.

Doubtless these views are correct, and when more is known by the laity of the venereal triad, there will be less of these diseases. Of the triad, gonorrhea should be selected as the prime subject for elucidation, for upon that the public, while most familiar, is most ignorant.

There is a curious contrast in the view taken by the public of gonorrhea and syphilis. The latter is the constant nightmare in the dream of pleasure. It is dreaded above all other diseases. It is a common experience for doctors to hear their patients express the most direful resolutions and threats of what they would do to themselves if they should "catch" syphilis. The public pictures the victim of the disease as a pariah covered with sores, his genital organs sloughing away, his bones rotting with disease.

On the other hand the estimate of gonorrhea is usually flippant, even cheerful. A case of gonorrhea is something of a toga virilis, necessary to the full development of manhood. It is viewed as something temporarily incommoding, a rather disagreeable discharge, with a remote contingency of a swelled testicle.

All the modern knowledge of the far-reaching effects of this disease is whistled down the wind. Impetuous youth has a wholesome fear of syphilis, but is smilingly incredulous over the seriousness of gonorrhea. It will be a long and hard campaign of education that will rectify this misconception and place gonorrhea upon the wall as a companion picture of syphilis.

By educational means venereal disease may be—it is possible—slowly eliminated ; but the animal craving that fosters its existence are as uneradicable and as uncontrollable as the promptings of hunger and thirst. To suppress the social evil, if that means whoremongering and adultery, is as wild a dream as ever theorists vexed themselves with to none effect.

It is one of the human frailties which is as much a part of man as his strength. The waters of the Dead Sea cover the vices of Sodom and Gomorrah and the Cities of the Plain are undistinguished dust, but the social evil has survived these rude shocks, and to-day draws strength from opposition. "'Tis true 'tis pity, and pity 'tis, 'tis true."

